# Reconfigurable chaos in electro-optomechanical system with negative Duffing resonators

**DOI:** 10.1038/s41598-017-05020-w

**Published:** 2017-07-06

**Authors:** Leisheng Jin, Yufeng Guo, Xincun Ji, Lijie Li

**Affiliations:** 1 School of Electronic Science and Engineering, Nanking University of Posts and Telecommunications, Nanking, Jiangsu 210023 China; 2 Multidisciplinary Nanotechnology Centre, College of Engineering, Swansea University, Swansea, SA1 8EN UK

## Abstract

Generating various laser sources is important in the communication systems. We propose an approach that uses a mechanical resonator coupled with the optical fibre system to produce periodic and chaotic optical signals. The resonator is structured in such a way that the nonlinear oscillation occurs conveniently. The mechanical apparatus in the configuration is the well known resonating system featured by the negative stiffness. The mechanical resonance is converted to reflected optical signal with the same dynamic properties as the mechanical oscillation, subsequently interacting with the optical signal within the optical fibre. The optical radiative force on the mechanical structure is also considered in the analysis. The coupled electro-optomechanical system has been analysed, and results show that the mechanical resonator has the capability to control the dynamics of the optical signal precisely. The system will have potential applications in tunable laser sources.

## Introduction

Thanks to the state-of-the-art fabrication techniques, in recent years various optomechanical systems using, e.g., microtoroids^1^, microspheres^2^, microdisks^3^, suspended mirrors^4^ and cavities with membrane in the middle^5^, have been fabricated and investigated theoretically and experimentally, opening up new possibilities in research ranging from fundamental physics, such as ground state cooling^6^ and quantum entanglement^7^, to practical applications, such as quantum-limited detection of forces and displacements^8^.

Chaos dynamics in optomechanics refers to the study of mechanism and/or dynamics of chaos phenomenon with respect to parameters including optical detuning, laser pumping, and overall damping rate^9^. This phenomenon has been widely studied and proven to be ubiquitous in conventional optomechanical systems^10–12^. For example, by fabricating a very high-Q factor microtoroid oscillator^1^, Carmon *et al*. have observed the chaotic motion of optical field experimentally when increasing laser pump to an certain extent. Very recently, Faraz Monifi *et al*.^13^ have experimentally demonstrated the chaos-induced stochastic resonance and chaos transfer between two optical fields in an optomechanical system. Beyond classical domain, L. Bakemeier *et al*. have analysed the route to chaos and period-bifurcation mechanism in the quantum regime of an experimentally accessible optomechanical system^14^. G. Wang *et al*. have claimed the transient chaos could be served as a resolution for explaining the phenomenon of quantum-classical correspondence breakdown^15^.

However, in these previously studied systems, because the nonlinearity is only originated from the interaction force *F*_*int*_, which is induced by photons inside of the optical cavity when changing their momentum and can be expressed as: ${F}_{int}=G\hat{x}\hat{a}$, where *G* is the coupling constant between mechanical displacement $\hat{x}$ and optical amplitude $\hat{a}$. One has to exert a large enough optical pumping or have a very high-Q factor mechanical resonator for generating chaos. This limits its use in systems such as random generator^16^ and secure communication^17^. Recently, hybrid electro-optomechanical systems (EOMS)^18, 19^, have been proposed for realizing a more controllable chaos in optomechanical systems. Instead of modulating optical pumping, one can alternatively manipulate the chaos in phonon interface. Typical examples are optomechanical systems that are coupled by an extra microwave LC resonator, in which the generated chaos can be modulated by electrical signals^20^.

**Figure 1:**
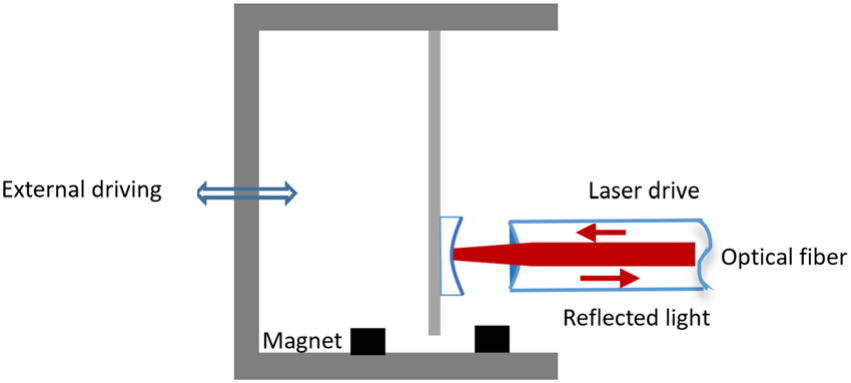
Schematic diagram of the proposed hybrid electro-optomechanical system.

In this work, we propose a new design of EMOS (see Fig. 1) by changing the mechanical oscillator in conventional optomechanical systems with a Duffing osccillator with a negative stiffness. Duffing oscillator with the negative stiffness has been experimentally realized by Ueda^21^ and is well-known for generating a controllable chaos^22, 23^. Here, we first combine this Duffing oscillator with the optical fibre cavity to investigate nonlinear dynamics in three modes: 1) Self-oscillation mode without the external driving from mechanical part. 2) Double-driving mode with the external driving added to mechanical part. 3) Parametrical-driving mode with an extra parametric pump added to mechanical part. It is revealed that chaos can only be generated in mechanical states of the self-oscillation mode while the optical fibre cavity functions as a frequency filter, both in the mechanical and the optical state chaos can be generated in the double-driving mode even with a very weak optical power pump. In the parametric-driving mode the generated optical chaotic signal can be manipulated from chaotic state to periodic state with a good accuracy. Detailed bifurcation and Melnikov analysis have been given to deeply understand the nonlinear behaviour and control mechanism. This new EMOS provides a new paradigm for studying nonlinear dynamics and exploring future on-chip MEMS based secure communication.

## Model Construction

The system shown in the Fig. 1 can be mathematically described by the Hamiltonian, which is

1 $$H={H}_{m}+{H}_{o}+{H}_{mo}+{H}_{d},$$

where *H*_*m*_ and *H*_*o*_ describe mechanical and optical mode, respectively, and *H*_*mo*_ represents the coupling between the two modes. These sub-Hamiltonians terms can be written as:

2 $${H}_{m}={\omega }_{m}({p}^{2}+{q}^{2})/\mathrm{2;}\,{H}_{o}={{\rm{\Delta }}}_{0}{a}^{\dagger }a;\,{H}_{mo}=-{G}_{o}{a}^{\dagger }aq;\,{H}_{d}=i\hslash (E{e}^{-i{\omega }_{0}t}{\hat{a}}^{\dagger }-{E}^{\ast }{e}^{i{\omega }_{0}t}\hat{a}),$$

where *ω*_*m*_ is the natural frequency of the mechanical mode. *q* and *p* are displacement and velocity of the mechanical mode, respectively. ${a}^{\dagger }$ and *a* are creation and annihilation operators associated with the optical mode, respectively. *G*_0_ and Δ_0_ represent the coupling strength between the mechanical and optical modes, and the laser detuning, respectively. The dynamics of the coupled system is governed by quantum Langevin equations^24^, which reads:

3 $$\partial \hat{O}/\partial t=i[\hat{H},\hat{O}]+\hat{N}-{\hat{H}}_{diss},$$

where $\hat{N}$ is the quantum fluctuation operator, ${\hat{H}}_{diss}$ denotes the dissipation, and $\hat{O}=p,q,a$ represents the operators of the coupled systems. The set of quantum Langevin equations is then given by:

4 $$\begin{array}{rcl}\dot{q} & = & {\omega }_{m}\,p\\ \dot{p} & = & -{\omega }_{m}q-{G}_{0}{a}^{\dagger }a-{\gamma }_{m}\,p+\xi \\ \dot{a} & = & -(\kappa +i{{\rm{\Delta }}}_{0})a-i{G}_{0}aq+E+\sqrt{2\kappa }{a}^{in},\end{array}$$

where *γ*_*m*_ and *κ* are the damping rate and decay rate of the mechanical resonator and cavity, respectively. The laser detuning is given by Δ_0_ = *ω*_*c*_ − *ω*_0_ with *ω*_*c*_ and *ω*_0_ are, respectively, the frequencies of the cavity mode and of the driving laser. *E* = *E*_0_ + *E*_1_ *cos*(Ω*t*) is external driving field, where Ω is the modulating term on the driving frequency *ω*_0_. *a* ^*in*^ is vacuum radiation input noise, which are stochastic processes and described by 〈*a* ^*in*^(*t*)*a* ^*in*,†^(*t*′)〉 = δ(*t* − *t*′). *ξ* is Hermitian Brownian noise operator characterized by $\langle \xi (t)\xi (t^{\prime} )+\xi (t^{\prime} )\xi (t)\rangle /2=$ ${\gamma }_{m}\mathrm{(2}\bar{n}+\mathrm{1)}\delta (t-t^{\prime} )$, where $\bar{n}=1/[\exp (\hslash {\omega }_{m}/{k}_{B}T)-1]$.

Now considering our model, as shown in Fig. 1, the mechanical beam is adjacent to two Magnets, which create distributed magnetic field force and couple acting on the beam. According to the established theory^21^, the force and couple can be given by: *F* = *M* · ∇*B* ^0^; *C* = *M* × *B* ^0^, where *B* ^0^ is magnetic field and *M* is magnetization induced by *B* ^0^. In this case, a magnetic energy potential can be found by using Galerkin approximation, which is written as: $W=-\mathrm{(1}/\mathrm{2)}\int M\cdot {B}^{0}d\nu$, and this potential is nonlinear in terms of beam’s modal amplitude *q* and can be expanded in a Tayler series in *q*, as:

5 $$W=\mathrm{(1/2)}\gamma {q}^{2}+\mathrm{(1}/\mathrm{4)}\beta {q}^{4}+\mathrm{(1}/\mathrm{6)}\eta {q}^{6}.$$

Combining the beam’s linear elastic force and the damping, a dynamical equation for describing the motion of the beam is arrived as

6 $$\ddot{q}+{\gamma }_{m}q+\beta q+\alpha {q}^{3}={f}_{0}cos({\omega }_{d}t\mathrm{)}.$$

Substitute equation (6) into equation (4), the coupled system will be studied. It should be noted that in the numerical simulation we will only focus on the classical realm, and the relation $O=\langle \hat{O}\rangle$ will be used, where $O\equiv (q,p,a,{a}^{\dagger })$. In addition, our model is actually the averaged version of quantum Langevin equations, as there are sufficiently large numbers of photons and phonons involved. Contrast to the quantum model driven by noise terms that comprise the vacuum noise and any thermal noise, it is the driving laser playing the main role. Therefore, quantum noises will be neglected in the following numerical study.

## Numerical Simulation

### Self-oscillating Mode

**Figure 2:**
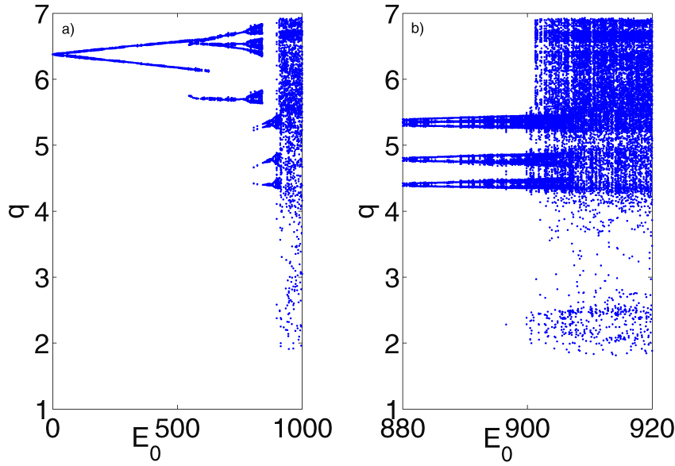
Bifurcation diagram of mechanical displacement *q* with varying *E*_0_. (**a**) is with a large *E*_0_ varying range and (**b**) is with an narrower range extracted from (**a**).

**Figure 3:**
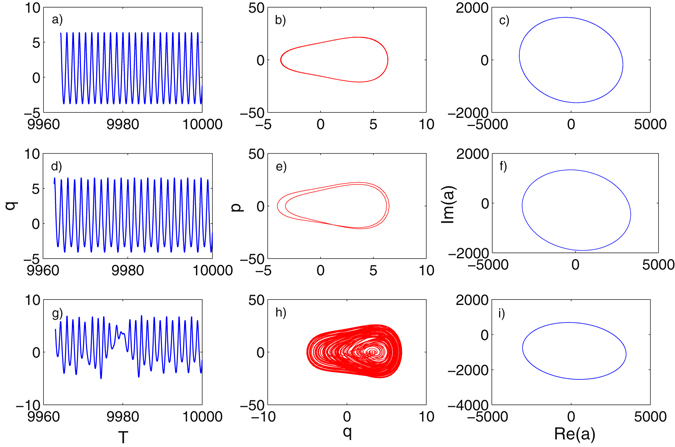
(**a**,**d** and **g**) are time series of *q* when *E*_0_ = 10, *E*_0_ = 200 and *E*_0_ = 980 with fixed *E*_1_ = 5000; (**b**),(**e**) and (**h**) are phase diagram between *q* and *p* of the mechanical state when *E*_0_ = 10, *E*_0_ = 200 and *E*_0_ = 980 with fixed *E*_1_ = 5000; (**c**),(**f**) and (**i**) plot the real part versus imaginary part of optical state *a* when *E*_0_ = 10, *E*_0_ = 200 and *E*_0_ = 980 with fixed *E*_1_ = 5000.

**Figure 4:**
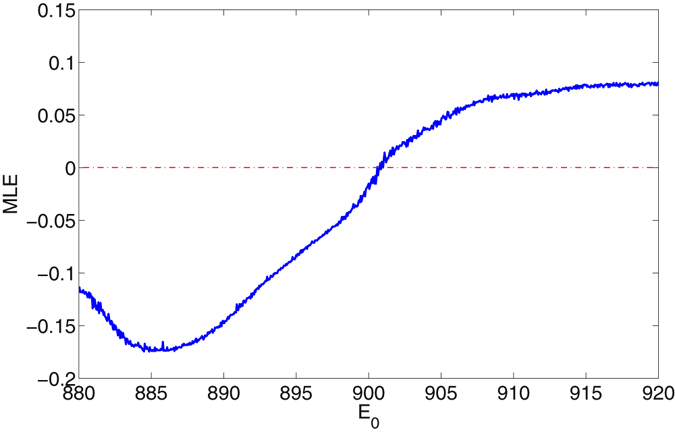
MLE of the system in self-oscillating mode with varying *E*_0_ ∈ [880, 920]; Red dot line stands for Zero-standard line.

First we study the case when there is no external driving exerted from the mechanical resonator. The parameters are taken as: *γ*_*m*_, *β* = 1, *α* = 1, *ω*_*d*_ = *ω*_*m*_, *L* = 100 mm^25^, *ω*_*m*_ = 1.4 × 10^5^ Hz, *κ*/*ω*_*m*_ = 0.22^9^, Ω = 2*ω*_*m*_ and Δ_0_/*ω*_*m*_ = 1. We have used the relation *T* = *ω*_*m*_ *t* in conducting the simulation. *G*_0_, the coupling constant between the mechanical mode and the optical mode, can be calculated by:

7 $${G}_{0}=\frac{{\omega }_{c}}{L}\sqrt{\frac{\hslash }{2{m}_{eff}{\omega }_{m}}},$$

where *ω*_*c*_ is frequency of the optical mode in cavity and given by *nc*/4*L* with *n* as the index of the cavity resonance of interest. *m*_*eff*_ is the effective mass of the resonator here taken to be 1 *ng*. In Fig. 2, we have calculated the bifurcation diagram of mechanical displacement *q* by taking optical driving term *E*_0_ as the varied parameter. As *E*_0_ varying from 0 to 1000 and with *E*_1_ fixed at 5000, the displacement *q* goes though periodic state and is finally bifurcated into chaotic state when *E*_0_ increased to 902. Specifically, as we can see, in Fig. 2(a), when the mechanical oscillation is in periodic state (0 < *E*_0_ < 902), it starts from period-1, then branches into period-2, and until period-n, with a down-turn occurred at *E*_0_ = 860 where it is seen that the amplitude of *q* is decreased and three main branches appear. In order to see the bifurcation of the state *q* from periodic and chaotic state, a more condensed-calculated bifurcation diagram is given in Fig. 2(b), where we can see how the *q* evolves into chaotic state with the amplitude of *q* changing non-regularly. When the mechanical state *q* is chaotic, the attractor region is bounded as we can see from Fig. 2(b). To have a clear picture of how the state *q* oscillates and evolves, time series of *q* in three typical phase (period-1 when *E*_0_ = 10, period-2 when *E*_0_ = 200 and chaotic state when *E*_0_ = 980) have been plotted in Fig. 3(a,d and g), where amplitudes of oscillating *q* are seen to be consistent with bifurcation diagram. Accordingly, the phase diagram of *q* in these three phases with their derivatives *p* are plotted in Fig. 3(b,e and h), in which phase trajectories further prove the periodic and chaotic state of mechanical mode. However, different from the dynamics of mechanical mode (*q*, *p*), *a* representing the optical mode in studied systems (equation (4)), exhibits a more tedious dynamics as it keeps oscillating in period-1 as *E*_0_ varying in the range set in Fig. 2, with only the oscillating amplitude changes slightly. This has been shown in Fig. 3(c,f and i) accordingly, where the imaginary and real part of the optical field *a* are plotted in pair indicating the period-1 state of the optical mode. The reason of this phenomenon is due to that the oscillation of mechanical mode, under the parameters setting, makes an ignorable effect on the optical resonance in the optical cavity, especially when the *E*_0_ is set to large values. The optical cavity is working as a filter that filters most of the oscillating modes of *q*. To further proves the chaotic state of the mechanical oscillation driven by the optical driving, we have also calculated the maximum Lyapunov exponent (MLE) of the coupled systems, an index used for signifying dynamical state of nonlinear dynamical systems (MLE > 0: chaotic; MLE < 0: periodical;). The results is shown in Fig. 4. It is seen the MLE is changing from negative to positive value at *E*_0_ = 902, indicating the mechanical mode is evolved at this point into chaotic state, which is consistent with the bifurcation calculation in Fig. 2(a) and (b).

### Double-driving Mode

**Figure 5:**
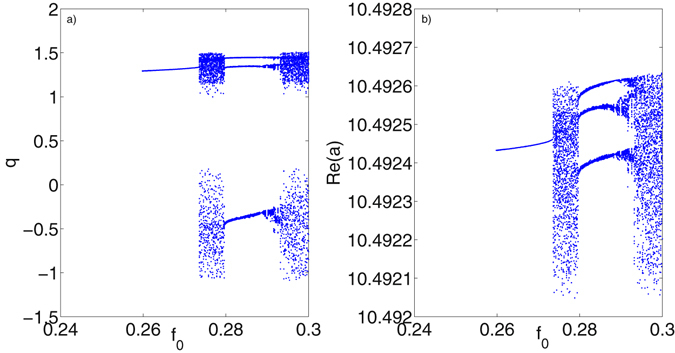
Bifurcation diagram of mechanical displacement *q* with varying *f*_0_ in (**a**) and bifurcation diagram of real part of optical state *a* with same range of varying *f*_0_ in (**b**).

**Figure 6:**
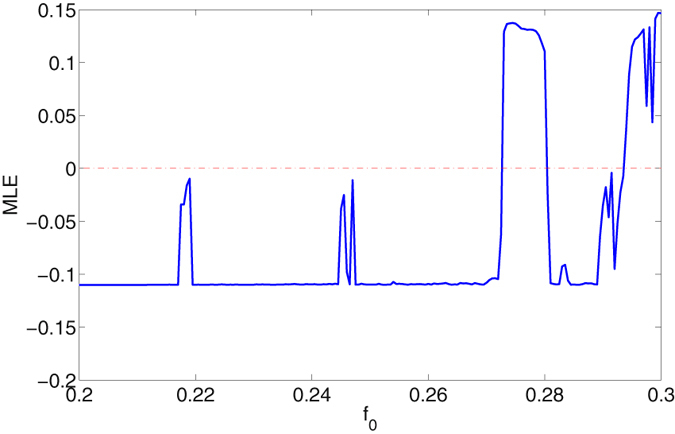
MLE of the system in double-driving mode with varying *f*_0_ ∈ [0.2, 0.3]; Red dot line stands for Zero-standard line.

**Figure 7:**
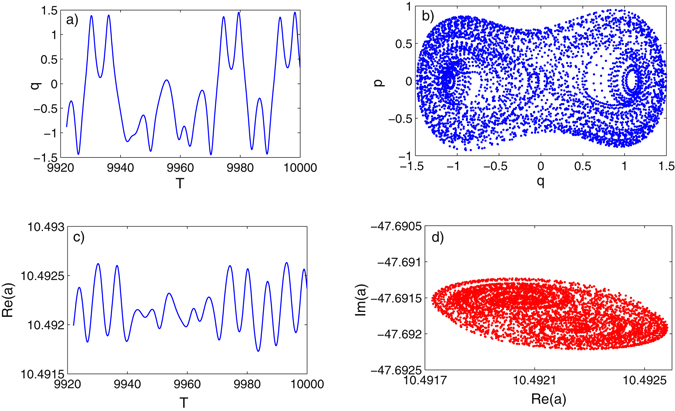
Time series of mechanical state *q* and optical state *a* (real part) in (**a**) and (**c**); Corresponding chaotic attractor of mechanical and optical mode in (**b**) and (**d**).

**Figure 8:**
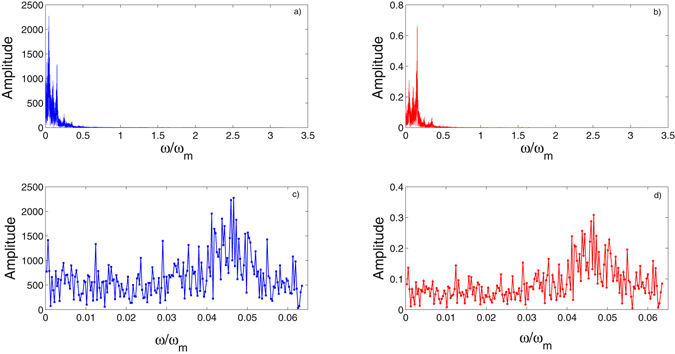
FFT analysis of the system when it is in double-driving mode and chaotic state. (**a**) and (**b**) are amplitude of mechanical and optical mode in frequency domain. (**c**) and (**d**) show a narrows frequency varying range.

In this section, we study the dynamics of the double-driving mode, i.e. when external drivers from the mechanical resonator and the optical driving are both exerted. In this mode, we can realize the dynamical regime where both the mechanical and optical variables enter into chaotic states. The parameters except for the optical driving terms *E*_0_ and *E*_1_ being fixed to 50 and 0, respectively, are unchanged. Likewise, we have first conducted a generalized bifurcation study of the mechanical mode *q* and the optical mode *a*, and the results have been presented in Fig. 5. It is seen from Fig. 5(a) and (b) that the two modes go through a similar dynamical route as the external driving strength *f*_0_ increased from 0.25 to 0.3. Specifically, both the mechanical amplitudes of *q* and optical field *a* (represented by the real part as the imaginary part has the same dynamics as the real part) are bifurcated from period-1 state abruptly into chaotic state at *f*_0_ = 0.273, and after a small dynamical window of chaotic state they are switched back into periodic state with the period-3. After this periodical oscillating range, the two modes are bifurcated to chaotic state again when *f*_0_ increased to 0.293. It can be concluded from the bifurcation analysis that the mechanical oscillation and optical filed at the double-driving mode interact with each other. It should be noted that the generated chaotic signal of optical state *a* is based on a very weak optical pump input (*E*_0_ = 50 and *E*_1_ = 0), which overturns the intuition that only large enough optical pump strength can generate a chaotic optical signal in optical cavity that is coupled with traditional Duffing resonator. The MLE of the coupled system in the double-driving mode has been calculated in Fig. 6, where it is seen the MLE becomes to be positive in the same range of chaotic states shown in bifurcation diagram Fig. 5, further proving the deterministic chaos. We have also plotted the time series of mechanical and optical states and their corresponding chaotic attractors in Fig. 7, which can be seen as a supplement proving deterministic chaotic states. To better understand the frequency components of the generated chaotic signals, a Fast Fourier transform (FTT) analysis when the coupled system is in chaotic state has been conducted. As shown in Fig. 8(a) and (b), the fluctuation of the amplitude of the mechanical and optical state in frequency domain synchronize with each other and the main frequency components of the chaotic signal exist at relatively low frequency range, i.e. *ω*/*ω*_*m*_ ∈ [0, 0.006 *ω*_*m*_]. In Fig. 8(c) and (d), enlarged diagram of Fig. 8(a) and (b) at the frequency range [0, 0.006 *ω*_*m*_] are given.

## Parametric Control Mode

### Results

**Figure 9:**
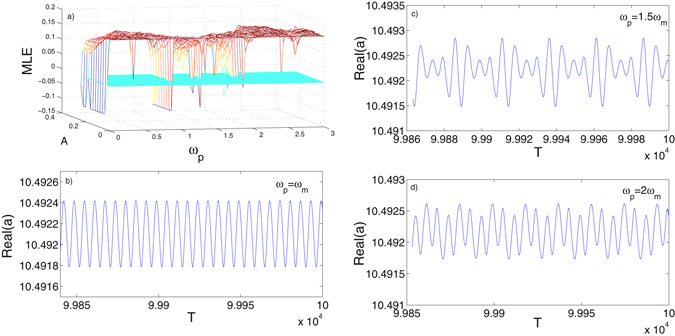
Controllability of parametric driving when setting *ω*_*p*_ = 1*ω*_*m*_ (**b**), *ω*_*p*_ = 1.5 *ω*_*m*_ (**c**) and *ω*_*p*_ = 2 *ω*_*m*_ (**d**). (**a**) is the plotting of MLE with varying parametric frequency and amplitude.

We have seen the deterministic chaos generated in the double-driving mode that is based on a small optical pump driving. Here in this section we study to manipulate the chaotic signal by utilizing the parametric driving. The mechanical resonator with parametric driving can be modelled as^23^

8 $$\ddot{q}+{\gamma }_{m}q+\beta q-Acos({\omega }_{p}+\phi )q={f}_{0}cos({\omega }_{d}t),$$

where *A* is amplitude of the parametric driving. *ω*_*p*_ and *φ* are frequency and phase of the parametric driving, respectively. Keeping all the parameters as same as in the section of double-driving mode when the system is in chaotic state, we investigate how the parametric frequency *ω*_*p*_ affects the chaotic signal generated in optical cavity. From the calculation of the time series of the optical signal *a*, it is found that the parametric frequency *ω*_*p*_ demonstrates a direct controllability on the chaotic signal. By setting *ω*_*p*_ as *ω*_*p*_ = *ω*_*m*_, *ω*_*p*_ = 1.5 *ω*_*m*_ and *ω*_*p*_ = 2*ω*_*m*_, *A* = 0.2 and *φ* = 0, we can manipulate the chaotic signal into period-2, period-1 and period-3 states, as shown in Fig. 9. We have also calculated the Lyapunov exponent of the system in parametric control mode in Fig. 9(a), in which we calculated the MLE of the EMOS with varying parametric frequency in (0–3 *ω*_*m*_) and varying amplitude in [0, 0.25] and parameters setting range to make MLE change from positive to negative can be detected.

### Analysis

To unveil why parametric driving has such a controllability on the generated chaotic signal in our coupled system, we have conducted Melnikov analysis of the system in the case when *ω*_*p*_ = 1. Other parameters are taken as: *γ*_*m*_ = 0.2, *β* = 1, α = 1, *A* = 0.2, *φ* = 0, *f*_0_ = 0.3 and *ω*_*d*_/*ω*_*m*_ = 1. First, the Hamiltonian of the mechanical resonator can be expressed as: $H=\mathrm{(1/2)}{\dot{q}}^{2}+\mathrm{(1/2)}\beta {q}^{2}+\mathrm{(1/4)}\alpha {q}^{4}$. The Hamiltonian is conserved and one can easily derive that it has two homoclinic orbits, which can be specially written as: ${{\rm{\Gamma }}}_{\pm }:({x}_{0}(t),{y}_{0}(t))=\pm \sqrt{2}sech(t),\mp \sqrt{2}sech(t)tanh(t)$. Here the *x*_0_ and *y*_0_ correspond the coordination of a specific point on the orbit. The Melnikov function of the system (equation (8)) is then obtained as:

9 $$M({T}_{0})={\int }_{-\infty }^{\infty }[{y}_{0}(\delta {y}_{0}+\gamma cos({\omega }_{d})(t+{T}_{0})+Acos{\omega }_{p}(t+{T}_{0})\cdot {x}_{0})]dt.$$

Substituting *x*_0_ and *y*_0_ in the equation (9), we can obtain *M*(*T*_0_) as:

10 $$M({T}_{0})=-\frac{4}{3}\delta \pm \sqrt{2}\gamma \pi \omega sech(\frac{\pi \omega }{2})sin(\omega {T}_{0})+A\pi {\omega }_{p}^{2}cech(\frac{\pi \omega }{2})sin({\omega }_{p}{T}_{0}\mathrm{)}.$$

**Figure 10:**
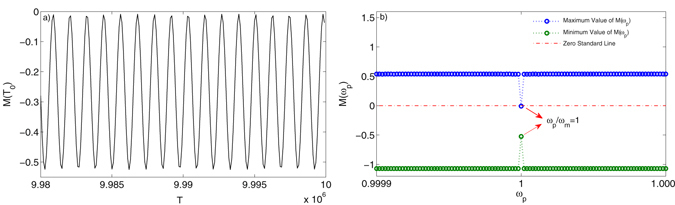
(**a**) is the *M*(*T*_0_) when the system is parametrically driven with *ω*_*p*_ = 1. (**b**) is the plotting of *M*(*T*_0_) with a very narrow varying range around *ω*_*p*_ = 1.

The *sech*(*t*), *cech*(*t*) and *tanh*(*t*) are all hyperbolic functions. The condition of *M*(*T*_0_) = 0 holding real roots is necessary for realizing chaos in the system. We have calculated *M*(*T*_0_) = 0 for a very narrow varying range of *ω*_*p*_, i.e. *ω*_*p*_ in [0.9999*ω*_*m*_, 1.0001*ω*_*m*_]. It is found that only when *ω*_*p*_ = *ω*_*m*_ the *M*(*T*_0_) does not have real roots. The result has been plotted in Fig. 10(a) and (b). To be more specific, In Fig. 10(a), the time series of *M*(*T*_0_) when *ω*_*p*_ = 1*ω*_*m*_ is given, where we can see the *M*(*T*_0_) does not have zeros. In Fig. 10(b), the maximum and minimum values of *M*(*T*_0_) with varying *ω*_*p*_ are plotted, where we can see that there is a sharp dip occurred when *ω*_*p*_ = 1 × *ω*_*m*_ and the maximum and minimum values of *M*(*T*_0_) do not cross the zero line meaning there is no real roots for *M*(*T*_0_) = 0. Therefore, when the parameter driving frequency is set to be 1*ω*_*m*_, the parametric driving is capable of manipulating the chaotic state into periodical state, which is consistent with the result shown in Fig. 9(c).

## Conclusion

To summarize, several laser chaos generation methods have been simulated using the setup assisted by a nonlinear mechanical resonator. It is found that the mechanical resonator plays the deterministic role in realizing the laser chaos, especially when it is designed to exhibit structural nonlinearity, where in this particular case, it is the negative stiffness introduced by the external parametric driver. The modelling results clearly demonstrated that the mechanical dynamics has the capability to manipulate the laser dynamics. The proposed method provides a solution to generate desired laser sources with microelectromechanical systems resonators.
